# Postoperative Maxillary Cyst: A Case Report

**DOI:** 10.4061/2010/810835

**Published:** 2010-11-04

**Authors:** Asiye Şafak Bulut, Celal Şehlaver, Alp Korkut Perçin

**Affiliations:** ^1^Department of Pathology, MESA Hospital, Yasam Caddesi no 5, Sogutozu, 06510 Ankara, Turkey; ^2^Department of Dental Diseases, MESA Hospital, Yasam Caddesi no 5, Sogutozu, 06510 Ankara, Turkey; ^3^Department of Ear, Nose & Throat, MESA Hospital, Yasam Caddesi no 5, Sogutozu, 06510 Ankara, Turkey

## Abstract

Postoperative maxillary cyst is a quite rare delayed complication of surgical intervention associated with maxillary sinuses. It occurs many years after surgery. This paper describes a 54-year-old woman presenting with swelling of left cheek for seven-years duration. The orthopantomograph revealed a unilocular cystic radiolucency with well-defined margins in left maxillary sinus. In the computerized tomography, the cyst had a sclerotic wall with bony condensations. Aspiration cytology revealed many neutrophil leukocytes. Cyst was drained and enucleated. Histopathologically, it had a fibrous wall with inflammation and focal reactive bone formation and lined by a respiratory-type epithelium. In the clinical history, it is learned that she had a maxillary sinus surgery 8 years ago and the diagnosis was made considering the clinical and histopathological findings.

## 1. Introduction

Postoperative maxillary cyst (PMC), which is also known as surgical ciliated cyst, postoperative paranasal cyst, or respiratory implantation cyst, was originally described by Kubo in 1927 [1]. It occurs up to 49 years after surgery associated with maxillary sinuses. Although it is quite rare in Western countries, it constitutes 20% of oral cysts in Japan. It presents as an expansile swelling of cheek or palate. Radiographically, it appears as a well-defined unilocular radiolucency in the maxillary sinus. It is lined by a respiratory-type epithelium, and this supports the theory suggesting that it results from the mucosa of maxillary sinus entrapped in the wound during closure or healing. Here, we report a case occurred 8 years after a maxillary sinus surgery.

## 2. Case Report

**Figure 1:**
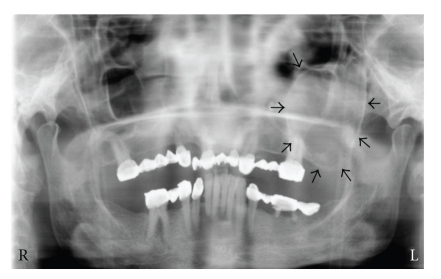
A unilocular well-defined radiolucency on orthopantomography.

**Figure 2:**
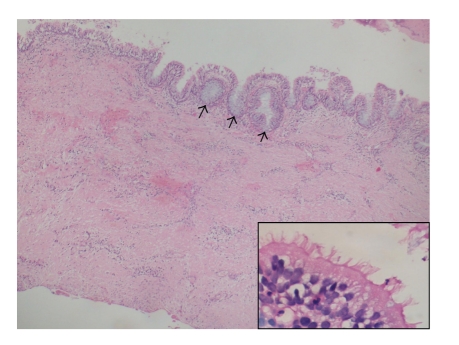
The thick fibrous wall of the cyst is lined by pseudostratified ciliated epithelium with goblet cells (arrows) (HE, original magnification is ×40, inset ×400).

A 54-year-old woman was admitted to our dental clinic suffering from repeating swelling of her left cheek during the past seven years. Physical examination revealed a mild bulk just under the left zygoma which was tender on palpation. The left buttress area was fluctuant and pus was draining from the posterior maxilla, giving the sense of an infected lesion which eroded the maxillary lateral wall. There was no evidence of pulpal or periodontal infection, neither clinically nor radiologically. On the orthopantomograph, there was a unilocular well-defined radiolucency in the left maxillary sinus region (Figure 1). Computerized tomography revealed the same lesion expanding the boundaries of the sinus through the surrounding soft tissue. Her medical history revealed a maxillary sinus surgery 8 years ago. Aspiration cytology revealed many polymorphonuclear leukocytes. The cyst was enucleated under local anaesthesia through a left Caldwell incision with some modifications. On macroscopic examination, it was thick walled with 4 cm diameter and had a smooth inner surface. In the microscopic examination, the wall was made up of thick fibrous tissue with focal reactive bone formation and widespread lymphocytic infiltration. The epithelium was pseudostratified ciliated epithelium with Goblet cells (Figure 2). No squamous metaplasia was observed. She recovered uneventfully and is being followed up regularly.

## 3. Discussion

A cystic lesion in the maxillary sinus is generally supposed to be a pseudocyst, but epithelium-lined cysts like mucoceles, odontogenic cysts, simple bone cysts, PMCs, fissural, and other nonodontogenic cysts can also be seen in this region. Pseudocysts represent focal accumulation of inflammatory exudate that lifts the epithelial lining of the sinus and the periosteum away from the underlying bone. Their histologic appearance is therefore that of normal or inflamed maxillary sinus lining, and there is no epithelium-lined cavity present beneath the sinus mucosa. Mucoceles are epithelium-lined, mucus-containing sacs. There are two mucocele types, primary and secondary. Primary mucoceles are mucous retention cysts whereas secondary mucoceles are caused by various conditions including chronic obstruction of the sinus ostia, mucosal inflammation, previous surgical procedures, benign and malignant lesions, chronic infection, or allergic disease [2].

PMC (or surgical ciliated cyst) is a kind of secondary mucocele caused by previous surgical procedures. It was firstly described by Kubo in 1927 [1], and many cases have been reported so far. As it constitutes 20% of all oral cysts in Japan, Japanese authors reported large series of cases [3–6], while there are case reports from Western countries [7–9]. There is only one case in a patient of African origin [10]. 

PMC can develop after midface osteotomies, traumatic tooth extraction, maxillary fracture, or a complication of Caldwell-Luc procedures. Many years may pass before the cyst is detected. Men are affected equally or somewhat more often than women, and the highest incidence occurs fourth to sixth decades of life. The clinical presentation ranges from asymptomatic to an indolent enlargement causing an aesthetic problem or acute swelling and pain, due to secondary infection. Some lesions can damage the boundaries of the sinus, producing perforation of the canine fossa, nasal wall, sphenopalatine wall, or orbital floor. Radiographically, it is presented as a unilocular or multilocular well-defined radiolucency; in some instances, it is surrounded by a zone of sclerosis. As it enlarges, sinus wall becomes thinned and eventually perforated. Gradually, the lesion expands beyond the original sinus boundaries. Their common histological feature is the ciliated respiratory-type epithelium. Squamous metaplasia may occur [11].

Treatment is enucleation or marsupialization of the cyst [12]. Few reports are available concerning the outcome of treated PMCs. K. C. Lee and N. H. Lee reported a recurrence rate of 20% for secondary mucoceles including PMCs [13].

The possible mechanism suggested by Kubo (1927 and 1933) is the entrapment of the sinus and/or nasal mucosa in the wound during closure. During the healing process, the sinonasal epithelium proliferates and creates the ciliated cyst.

Among the cysts considered in the differential diagnosis, pseudocysts do not have an epithelial lining. Mucous retention cysts (primary mucoceles) are small, thin-walled cysts and are generally found incidentally. Odontogenic cysts can expand to the maxillary sinuses but they are lined by stratified squamous epithelium and contain keratinous material [14, 15]. Fissural and other nonodontogenic cysts are lined by stratified squamous or respiratory-type epithelium or a combination of both. But they are located outside the maxillary sinus. Simple bone cyst usually occurs in the young patient and it is rarely located in the maxilla. It forms a sharply outlined unilocular radiolucent mass radiographically but it is not lined by epithelium.

Here, we reported a PMC occurred 8 years after a maxillary sinus surgery. The proliferation of the entrapped sinonasal mucosa in the defective area may form such a cyst during the healing phase. We believe this case exhibits another example of this possible mechanism.
